# Personalized robots for long-term telerehabilitation after stroke: a perspective on technological readiness and clinical translation

**DOI:** 10.3389/fresc.2023.1329927

**Published:** 2024-01-08

**Authors:** Yanhuan Huang, Bibo Yang, Thomson Wai-Lung Wong, Shamay S. M. Ng, Xiaoling Hu

**Affiliations:** ^1^ Department of Biomedical Engineering, Hong Kong Polytechnic University, Kowloon, Hong Kong SAR, China; ^2^ Department of Rehabilitation Sciences, Hong Kong Polytechnic University, Kowloon, Hong Kong SAR, China

**Keywords:** stroke, long-term telerehabilitation, personalized robot, clinical translation, technological readiness

## Abstract

Stroke rehabilitation, which demands consistent, intensive, and adaptable intervention in the long term, faced significant challenges due to the COVID-19 pandemic. During this time, telerehabilitation emerged as a noteworthy complement to traditional rehabilitation services, offering the convenience of at-home care delivery and overcoming geographical and resource limitations. Self-help rehabilitation robots deliver repetitive and intensive physical assistance, thereby alleviating the labor burden. However, robots have rarely demonstrated long-term readiness for poststroke telerehabilitation services. The transition from research trials to general clinical services presents several challenges that may undermine the rehabilitative gains observed in these studies. This perspective discusses the technological readiness of personal use robots in the context of telerehabilitation and identifies the potential challenges for their clinical translation. The goal is to leverage technology to seamlessly integrate it into standard clinical workflows, ultimately enhancing the outcomes of stroke rehabilitation.

## 1 Introduction

Over the past three years, the world has grappled with the COVID-19 pandemic, prompting a significant shift in healthcare delivery (1). This global crisis accelerated the adoption of digital solutions and ushered in a new era in healthcare, with personalized rehabilitation now a vital component of long-term healthcare (2). The widespread implementation of quarantine, social distancing, and lockdown measures during the pandemic underscored the limitations of traditional healthcare systems while emphasizing the need for tailored healthcare approaches. Relying on frequent in-visit and face-to-face therapeutic interventions in hospitals and clinics has become increasingly impractical for both inpatient and outpatient care. Long-term stroke rehabilitation necessitates consistent, intensive, and responsive intervention, which was exceptionally challenging to deliver via traditional means during the pandemic (3).

Amid the rapid advancements in cyber-physical technologies driving digital economies across various industries, there is an urgent need to accelerate progress in long-term healthcare automation leveraging existing digital infrastructure, such as artificial Internet of things (AIoT) and data technology (DT) (4). Telerehabilitation has emerged as a valuable addition to traditional rehabilitation services, combining the convenience of in-home care delivery with traditional therapist-to-patient interaction, which effectively overcomes geographical limitations, resource shortages, and the risk of infectious disease transmission, especially during the COVID-19 pandemic (5). Meanwhile, its value extends far beyond the constraints of COVID-19 or similar epidemics, because it is a cost-effective, time-saving, flexible, and alternative service that is suitable for long-term monitoring, empowering patients to take an active role in managing their rehabilitative progress. The potential for remote monitoring to revolutionize rehabilitation delivery offers a glimpse into a more connected and proactive approach to healthcare.

Rehabilitation robots and virtual reality (VR) are two major tools for delivering telerehabilitation. Despite VR's immersive and interactive rehabilitative capacities, robotic rehabilitation manifests benefits including substantial physical engagement on target muscles, enhanced support for severely impaired patients, extensive data acquisition, and safeguards for physical stability, thereby substantiating their pivotal role within the telerehabilitation paradigm. However, the development of rehabilitation robots has lagged behind digital fields. Most are designed for use within healthcare facilities, relying heavily on healthcare professionals. Furthermore, the current robot designs lack the precision required to achieve optimal neuroplasticity during post-stroke rehabilitation, resulting in less effective outcomes compared with manual interventions (6). Challenges in telerehabilitation are primarily linked to the independence and compliance of individual stroke patients when professional supervision is not readily available, and the effectiveness of remote supervision and follow-up by professionals. Managing rehabilitative progress in a remote setting poses additional difficulties. Unfortunately, robots have seldom demonstrated feasibility for long-term post-stroke telerehabilitation with the required effectiveness. This perspective explores the technological readiness of robots for personal use in telerehabilitation and highlights potential challenges in their translation to clinical practice.

## 2 Robots for personalized rehabilitation

### 2.1 Rehabilitative effectiveness

Poststroke motor restoration involves relearning sensorimotor processes through intensive and repetitive activation of target neuromuscular pathways in the affected limb to achieve rehabilitative neuroplasticity. This process requires not only voluntary motor effort (VME) originating from the motor cortex to target muscles with minimum compensatory movements (7) but also simultaneous feedback via specific somatosensory pathways for precise motor control. Notably, significant motor improvements are possible even in the chronic stage of recovery (8). However, current robotic solutions face the following challenges that impede their rehabilitative effectiveness.

#### 2.1.1 Voluntary effort/intention control

The current robotic controls in rehabilitation face challenges in effectively promoting maximum voluntary motor effort (VME) through the intact ipsilesional neuromuscular pathway. Two primary strategies have been adopted: (1) central-intention-driven strategy, which relies on mental activities such as movement preparation detected by electroencephalography (EEG) to control robots via brain-computer interfaces (BCI) based on motor imagery (MI); (2) peripheral-effort-driven strategy, where signals from kinetic/kinematic/muscular measurements, such as electromyography (EMG), in the residual limb are used for robotic assistance (9). However, using BCI-MI alone, without simultaneous muscular effort generation, has shown limited effectiveness in stroke motor restoration, similar to conventional care approaches (10). MI primarily involves preparing for VME rather than generating it, which requires direct excitation from the motor cortex to induce muscle contraction. Moreover, peripheral-effort-driven robots have not demonstrated significant advantages over human therapists, even with higher repetition rates. This is mainly due to difficulties in accurately distinguishing VME from muscle activations in the residual limb, which can be distorted by poststroke muscle discoordination and involuntary muscle contractures caused by spasticity (11). Spasticity arises from overactivity in the spinal cord and brainstem's alpha motor neurons due to the loss of descending input from the cerebral cortex and basal ganglia (12). The presence of spastic muscle activations can inadvertently drive a robot without true VME, resulting in limited rehabilitative effectiveness (13). While methods such as sample entropy have been suggested to minimize spastic EMG signals by assuming that voluntary EMGs exhibit higher entropy than spastic EMGs, these methods lack direct cortical confirmation to validate their accuracy (13). Because VME was not precisely recruited, both control strategies triggered alternative neuromuscular pathways and resulted in compensatory neuroplasticity with inadequate rehabilitation. A more precise representation of cortically originated central-to-peripheral VME, which is capable of enhancing ipsilesional neuroplasticity in the descending track, is desired in personalized robot control design for stroke rehabilitation.

#### 2.1.2 Guided neuroplasticity during the rehabilitation

Observations during spontaneous motor recovery have highlighted that neurocircuitries with a cortical center, primarily located in the ipsilesional sensorimotor cortex, connecting to peripheral muscles for contralateral control, tend to yield superior motor outcomes compared with those with centers in the contralesional hemisphere (14). This advantage arises from the greater number of afferent/efferent neural pathways for contralateral control during movement, in contrast to the relatively few ipsilateral neural tracts (15). Furthermore, the ipsilateral neural pathways primarily target proximal joint muscles, such as the shoulder and elbow, with relatively less emphasis on the wrist and hand (16, 17). Dependence on contralesional neuroplasticity for motor recovery often results in extreme compensatory movements in proximal joints and the development of learned nonuse in wrist and hand muscles (18). Consequently, there is an urgent need for robotic interventions focused on facilitating maximum ipsilesional motor control while minimizing reliance on cortical and muscular compensation.

#### 2.1.3 Regulation of somatosensory pathway

Integration of precise and effective somatosensory input into muscles is essential for enhancing targeted afferent pathways. A hybrid robot that combines neuromuscular electrical stimulation (NMES) and mechanical actuation in a hybrid robot, known as NMES-robot, has demonstrated greater effectiveness compared with using NMES or mechanical assistance separately. This combination is especially beneficial to distal joint rehabilitation (19). This is because the assistive force helps a paretic limb achieve greater kinematic accuracy in gross joint motions, while NMES can directly evoke the contraction of habitually unused muscles by depolarizing both muscular motor and sensory neurons to facilitate the rehabilitative neuroplasticity for individual muscles (20). Despite advancements in upper limb rehabilitation, there remains a persistent lag in improving wrist-hand motor function compared with the progress made in shoulder and elbow rehabilitation. This disparity persists even when the NMES-robots were only applied to the distal joints (21, 22). The primary reason for this discrepancy is that current robotic control mechanisms do not effectively stimulate ipsilesional neuroplasticity to reduce the need for proximal compensation. Furthermore, they fail to engage targeted closed-loop neurocircuits for integrated motor and sensory responses, as discussed in Section 2.1.1.

### 2.2 Readiness for personal use

Personalized poststroke robots for long-term telerehabilitation, which require minimal professional assistance and remote supervision, can effectively support repetitive and intensive physical training when there is a shortage of professional manpower. These robots have the potential to complement traditional center-based and in-person outpatient services (23). The ideal telerehabilitation system should be user-friendly for nonprofessionals in unconventional environments, such as patients or caregivers at home, or even outdoors. They should prioritize safety, even in the event of a system malfunction, and be affordable for most stroke survivors (24). In this regard, the majority of current robots are not yet adequately suited for telerehabilitation. Most robotic systems used in clinical services with proven rehabilitative effectiveness are large and require professional operation in institutional environments (25). While there have been some developments in wrist–hand training systems for potential home use (26), few have been validated for their feasibility in telerehabilitation through trial studies. Despite technological advancements, developing a system that can mimic the sensitive, adaptive, and precise physical assistance that a trained human therapist can provide at home remains exceptionally challenging. Therefore, the focus should be on inventing robots that can effectively adapt to varying environmental conditions and patient needs in telerehabilitation.

## 3 Challenges in clinical translation

Personalized robots designed for telerehabilitation have demonstrated promise in improving motor function and supporting stroke recovery in research trials. These robots hold the potential to enhance the accessibility, convenience, and flexibility of clinical services when translated into general practice. However, during this translation process, various challenges may emerge that could undermine the rehabilitative gains observed in the trials. Common concerns revolve around device safety, usability, and ease of use. In addition, obstacles may arise in ensuring patients' readiness for independent training at home and in equipping therapists with the expertise to provide effective remote supervision for timely support (27). Several key challenges encountered during translation are discussed below.

### 3.1 Professional acceptance

One significant challenge during the translation process is the reluctance of clinical professionals to fully embrace the technical aspects, including training protocols, support schemes, and neuroimaging metrics. In actual clinical settings, protocol compliance can be hard to enforce, and flexibility tends to increase. Clinical therapists may be inclined to incorporate their established clinical routines into standard practice rather than strictly adhering to the research protocols that yielded successful outcomes in trials (28). In practical clinical services, where extended hospital stays are constrained by limited healthcare facilities, therapists often employ support strategies to complete limb tasks, regardless of the degree of compensatory motions, to expedite discharge from the hospital. These compensatory strategies can help stroke survivors adapt to their functional limitations, develop alternative methods for task performance, and achieve independence in activities of daily living (ADLs) during the early stages of stroke recovery (29, 30). However, this approach of employing compensatory strategies can lead to a condition known as “learned nonuse” of distal joints after stepping into chronic stroke, significantly limiting the potential for further motor recovery in these distal joints (31). In contrast, laboratory trials often adopt support-as-necessary strategies with the ultimate goal of enabling stroke patients to perform tasks independently, without assistance (32). As discussed by Qing et al. (33), the different support schemes used in laboratory trials and clinical services resulted in varied training outcomes with the same robotic device. Notably, the clinical group achieved less improvement in functional recovery and shorter long-term maintenance. Furthermore, the low acceptance of technical details, such as mathematical parameters and neuroimages, by clinical practitioners can pose difficulties in effectively integrating these metrics into clinical practice (34). These metrics typically require interpretation by experienced specialists with engineering backgrounds, which can act as barriers to their widespread adoption and use in routine clinical settings.

### 3.2 Education

The successful implementation and utilization of telerehabilitation requires a shift in the delivery of rehabilitation services from traditional in-person interactions to remote interactions, and education plays a crucial role for both professionals and users in this transition (35, 36). Healthcare professionals involved in telerehabilitation need appropriate training and skill development to effectively deliver these services. They must acquire knowledge of telecommunication technologies, virtual platforms, remote monitoring tools, and specific equipment used in telerehabilitation. Providing comprehensive training programs and continuous professional development opportunities is essential for healthcare professionals to gain the necessary skills for providing high-quality telerehabilitation services. In addition, professionals must adapt their clinical skills, assessments, and interventions to fit the remote environment. This adaptation may involve the acquisition of new communication strategies, ensuring patient engagement and active participation, and optimizing the use of technology to achieve therapeutic goals.

Users' participating in telerehabilitation programs, including patients, their families, or caregivers, should be equipped with the knowledge and support necessary to use telecommunication tools, understand the objectives of telerehabilitation, and actively engage in the programs. They need guidance in setting up and using equipment, resolving technical issues, and adhering to treatment plans. Providing patient-focused educational resources and ongoing support can empower patients in their telerehabilitation programs. However, education supervised by therapists is often insufficient, due to the shortage of resources in Hong Kong public rehabilitation centers, where each teaching session typically only lasts 30 min as an example (37). In contrast, the literature suggests that operators in a lab setting averagely offer one-on-one instruction lasting 60–90 min (33, 38). Inadequate professional education during these sessions can greatly reduce the usability and effectiveness of robotic devices when used at home by patients.

### 3.3 Quality of patient–operator interaction

The success of robot-assisted telerehabilitation hinges significantly on the quality of patient-operator interaction, which includes professional guidance, timely feedback, and interaction duration (39). Neurologically impaired patients frequently encounter obstacles, such as unfamiliarity and psychological resistance, when adopting new techniques, such as using robots and participating in telerehabilitation settings. Their confidence and willingness to embrace new approaches are typically lower than those of individuals without impairments (40). Ensuring high-quality patient-operator interactions can help address this challenge, leading to improved training outcomes by familiarizing patients with robot-assisted training, boosting their confidence, and engaging them in self-help telerehabilitation. In addition, the trust that patients place in the operator can significantly enhance their training experience during a new treatment. In the current clinical service, the quality of interaction between patients and operators was reported to be restricted due to limited interactive time, which led to delayed feedback, as discussed in Section 3.2 (33). The constrained schedule of public clinical services posed challenges for therapists in providing timely feedback, compounded by the fixed feedback times typically falling within regular business hours. The absence of immediate support and feedback during home sessions could potentially negatively impact patient engagement and motivation.

## 4 Future directions

Based on the concept of personalized robots for long-term telerehabilitation after stroke, several promising avenues for technological readiness and clinical implementation have emerged. First, the present technology landscape necessitates the development of robots equipped with enhanced motor control, adept at tailoring their functionality to the specific requirements of individual patients, and more adaptable to diverse environments. Ideally, these robots should be lightweight, compact, and portable, providing real-time feedback, greater training environment flexibility, and remote supervision without unduly taxing healthcare professionals. It should also be noted that striking a balance between the sophistication of technological advancements and the user-friendliness for patients of diverse educational backgrounds is also a crucial determinant in fostering the broad acceptance of remote personalized robotic systems for long-term telerehabilitation. Enhanced emphasis could be placed on simplifying and visually portraying user interfaces as well as feedback mechanisms in a comprehensible manner, such as employing color-coordinated indicators or simplistic directives. Accessibility of these advanced telerehabilitation systems to a wider demographic, independent of their educational attainment, could be augmented through personalized support provisioned by professionals, coupled with multimodal instructional methods including video tutorials, infographics, and unambiguous language instructions.

Beyond leveraging state-of-the-art technology to lessen the need for professional intervention via remote monitoring, the technological preparedness of post-stroke personalized rehabilitation robots should also focus on the provision of tailored adaption and autonomous theranostics, congruent with the patient's unique requirements. Technological readiness should prioritize enhancing robot autonomy through cutting-edge artificial intelligence (AI) technology, which can learn from individual patient data, e.g., EMG (41), EEG (42), electrical impedance myography (43), etc., enabling the adaptation of more personalized telerehabilitation and offering automatic theranostics over long-term service. It presents an economical and timely method for bridging the gap between professionals and patients for diagnosis, treatment, and follow-up, which is unconstrained by geographical limitations. For instance, a self-help rehabilitation robot integrated with point-of-care Internet of Things (IoT) systems can facilitate this vision.

Considering the shift in clinical practice, it is essential to align technological advancement with clinical validation and conduct further feasibility testing to transition telerehabilitation programs into routine clinical services. Furthermore, healthcare professionals should be encouraged to stay updated on the latest advancements in psychology, robotics, and even AI to provide comprehensive care and improve patient outcomes. Finally, improving the efficiency of operator supervision can boost the quality of patient-operator interactions. This involves equipping patients or caregivers with the necessary skills for more independent self-help sessions.

## 5 Conclusions

While increasing evidence supports the effectiveness of robotic training in poststroke telerehabilitation, the readiness of robots for personal use and their integration into real clinical settings remains a challenge. Overcoming these challenges necessitates a collaborative approach involving researchers, clinicians, engineers, policymakers, and stroke survivors themselves. To address these issues, it is crucial to pursue ongoing research, conduct rigorous clinical trials, garner support from health policymakers, and drive technological advancements. These steps will enable the successful incorporation of robotic training into routine clinical practice for stroke rehabilitation. Ultimately, the goal is to leverage robotics and automation techniques in conjunction with traditional therapy to enhance the effectiveness, personalization, and accessibility of rehabilitation.

## Data Availability

The original contributions presented in the study are included in the article/Supplementary Material, further inquiries can be directed to the corresponding author.
